# Human Protein Z as the Second Known Heme‐Binding Protein from the Endogenous Blood Coagulation Inhibitor System

**DOI:** 10.1002/cbic.202500636

**Published:** 2025-11-27

**Authors:** Paula Lindemann, Marie‐T. Hopp

**Affiliations:** ^1^ Bioorganic Chemistry, Chemistry Department Institute for Integrated Natural Sciences University of Koblenz Universitätsstraße 1 56070 Koblenz Germany

**Keywords:** blood coagulation, heme, heme–protein interactions, peptides, protein–protein interactions

## Abstract

Protein Z (PZ) is a vitamin‐K‐dependent glycoprotein that serves as an anticoagulant cofactor in blood. Although it is homologous to the serine proteases of blood coagulation, PZ has no enzymatic activity. It binds to the PZ‐dependent protease inhibitor and supports inactivation of factor Xa. In addition, an inhibitory effect on thrombin is described. Clinically, changed PZ levels increase the risk for thrombosis. The related activated protein C (APC) is described as the first anticoagulant protein that binds heme under hemolytic conditions leading to its inhibition. However, the network of inhibitors of blood coagulation is still underexplored with respect to their role in heme‐triggered effects and the regulation thereof. PZ seems to be an interesting candidate in this context due to its homology to APC. Using PZ‐derived peptides as models for potential heme‐binding sites in PZ together with in silico studies, one specific heme‐binding site in PZ is identified. Binding studies on protein level demonstrate binding characteristics similar to APC. Finally, the inhibitory effect of PZ toward thrombin is increased in the presence of heme, providing also insights into a potential functional consequence of the PZ–heme interaction. In addition, the anticoagulant function of PZ is dampened in the presence of heme in an aPTT‐based clotting assay, suggesting a tendency toward heme‐induced prothrombotic actions. In future, this will support mapping the diverse effects of heme within blood coagulation.

## Graphical abstract

Protein Z is a vitamin K‐dependent anticoagulant with elusive molecular functions. Herein, it is shown that heme binds to protein Z and induces conformational changes. One histidine‐based heme‐binding motif is identified. High heme‐binding affinity and functional effects are demonstrated on protein level. These findings shed new light on the structural and functional roles of protein Z in hemolysis‐driven coagulation disorders.

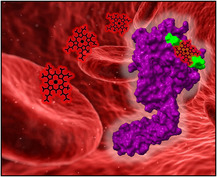

## 1 Introduction

Intravascular hemolysis, the premature rupture of red blood cells and subsequent release of hemoglobin into blood plasma, displays a pathological event with profound implications for vascular homeostasis.^[^ ^1^ ^,^ ^2^ ^]^ The heme moiety in hemoglobin rapidly oxidizes, thus leading to its release from the globin chains. While hemoglobin and heme levels are usually tightly regulated by scavenger proteins, the heme‐binding capacity of these proteins becomes overwhelmed in severe hemolytic conditions, such as in patients suffering from β‐thalassemia, sickle cell disease paroxysmal nocturnal hemoglobinuria, or hemolytic transfusion reactions, which leads to intravascular accumulation of labile heme.^[^ ^3^, ^4^, ^5^ ^]^ In this context, concentrations in the high micromolar range have been reported.^[^ ^4^ ^,^ ^6^ ^,^ ^7^ ^]^ Excess labile heme, however, contributes to oxidative stress, endothelial activation, proinflammatory reactions, and a strong prothrombotic state that exacerbates vascular dysfunction and tissue injury.^[^ ^8^, ^9^, ^10^, ^11^ ^]^ Although a direct interference of heme within the blood coagulation system was suggested already in 1911 when heme injections caused extravasating bleeding in guinea pigs and rabbits,^[^ ^12^ ^]^ direct interactions of heme with the blood clotting components were only proposed in 1983.^[^ ^13^ ^]^ Until today, coagulation factor VIII (FVIII) and fibrinogen are the only clotting factors discovered as heme‐binding proteins so far.^[^ ^14^, ^15^, ^16^, ^17^ ^]^ FVIII binds up to seven heme molecules with very high affinity (*K*_D_ of ≈2–13 nM) and is inhibited in its cofactor function supporting a bleeding tendency of this interaction.^[^ ^14^ ^,^ ^17^ ^,^ ^18^ ^]^ In contrast, fibrinogen binds heme with rather low affinity (*K*_D_ of ≈3 µM) and its cross‐linking is promoted by heme, tending toward prothrombotic consequences.^[^ ^15^ ^,^ ^16^ ^]^ Furthermore, activated protein C (APC) is the only known endogenous blood coagulation inhibitor demonstrated to bind heme so far.^[^ ^19^ ^]^ APC is a vitamin K‐dependent serine protease that plays a central role in maintaining the delicate hemostatic balance. Upon binding of one to two heme molecules with high affinity (*K*_D_ of ≈400 nM), APC is inhibited in its anticoagulant function by heme. These results suggested a novel mechanism through which heme may dysregulate coagulation, by impairing natural anticoagulant systems. Up to date, APC remains the sole documented member of the anticoagulant system with a capacity for heme binding. Given the complexity and redundancy of blood coagulation regulation, it is plausible that other anticoagulant proteins may also interact with heme under hemolytic conditions. Protein Z (PZ), a relatively understudied vitamin K‐dependent glycoprotein, emerges as a compelling candidate in this context.

PZ is a cofactor for the PZ‐dependent protease inhibitor (ZPI), a serpin, which primarily acts as an inhibitor of activated factor X, thus reducing thrombin generation.^[^ ^20^, ^21^, ^22^ ^]^ Though structurally homologue to other coagulation factors, PZ is catalytically inactive due to a lack of the critical serine residue in its active site which would be essential for protease activity.^[^ ^23^ ^]^ Thus, its function relies on its interaction with ZPI via its peptidase‐like domain (*K*_D_ of ≈1 nM^[^ ^20^ ^,^ ^24^ ^]^) and its interaction with phospholipid surfaces via its Gla domain (*K*_D_ of ≈48 µM^[^ ^25^ ^]^). In this context, different roles of PZ were postulated, including a function as a plasma carrier for ZPI, as an assistant for the immobilization of ZPI to the membrane as well as a mediator for the stereospecific interaction between ZPI and FXa.^[^ ^26^ ^]^ In particular, the Gla domain of PZ supports fast association of PZ with FXa on the membrane, which, in turn, accelerates inhibition of FXa by ZPI.^[^ ^27^ ^]^ Furthermore, direct binding to thrombin with a *K*_D_ of ≈110–150 nM has been reported that leads to a slight inhibition of thrombin's activity.^[^ ^28^ ^]^ Interestingly, alterations in PZ levels have been reported in patients with β‐thalassemia.^[^ ^29^ ^]^ However, reports on PZ levels under hemolytic conditions are generally rather rare. Low levels of PZ were observed in patients with PZ deficiency as well as in proinflammatory states, which has been associated with a predisposition to venous thrombosis.^[^ ^30^, ^31^, ^32^ ^]^ Although contradictory, also high levels of PZ have been reported to increase the risk for prothrombotic states, such as ischemic stroke.^[^ ^33^ ^]^ This mysterious and still underexplored nature of PZ together with its structural homology with APC depicts PZ as another potential heme‐binding protein in the system of endogenous blood coagulation inhibitors.

Herein, we demonstrate specific heme binding to human PZ with a similar heme‐binding affinity as APC. Through a combination of biochemical, biophysical, and bioinformatic approaches, we characterize the heme‐binding capacity of PZ, analyze potential heme‐binding motifs, assess the structural features that may be induced by this interaction, and explore the functional consequences of heme binding to PZ. The results extend the current understanding of how hemolysis perturbs coagulation homeostasis, not only through heme binding and regulation of clotting factors but also through the modulation of endogenous anticoagulants.

## 2 Results and Discussion

### 2.1 PZ Binds Heme

**Figure 1:**
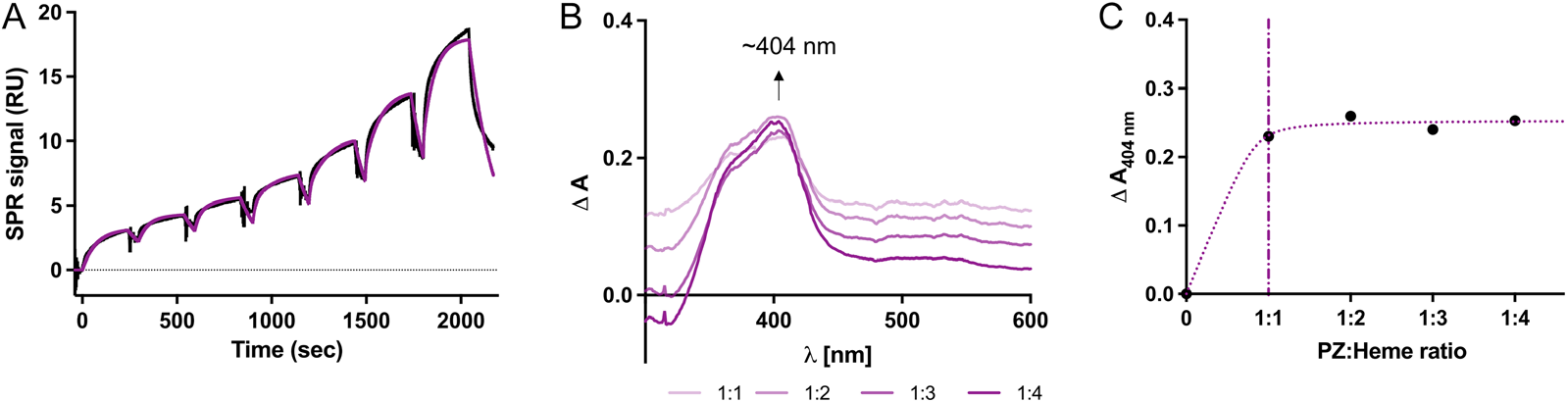
PZ is a heme‐binding protein. A) The single‐cycle kinetic SPR analysis of the interaction of heme (187.5 nM–12 µM) with PZ revealed a high affinity interaction with a *K*_D_ of 123 nM and additional unspecific binding with a *K*_D_ of 8.57 µM (kinetic global fit in purple). The observed fast association (*k*_a_ of 4.73 × 10^4 ^M^−1 ^s^−1^) and dissociation (*k*_d_ of 5.83 × 10^−3 ^s^−1^) is common for transiently heme‐binding proteins. B) UV/vis titration of heme (90 µM) to PZ (10 µM) in ratios from 1:1 to 1:4 PZ:heme support heme binding to PZ as evident by a Soret band shift to ≈404 nm. The difference spectra of the heme–PZ complex in different heme:protein ratios (1:1–1:4) are shown. C) Already at a ratio of 1:1, PZ was saturated with heme, indicating the presence of one specific heme‐binding site in PZ.

To investigate whether and to which extent heme interacts with PZ, SPR and UV/vis spectroscopic techniques were employed. SPR analysis revealed a high heme‐binding affinity for PZ (*K*_D1_ of 123 nM) (**Figure** **1**A). As such, its heme‐binding affinity is less strong than the affinity of FVIII for heme (*K*_D_ ≈ 2–13 nM),^[^ ^17^ ^]^ stronger than the one reported for fibrinogen (*K*_D_ ≈ 3.3 µM)^[^ ^15^ ^]^ but very similar to the one for the related protein APC (*K*_D_ ≈ 400 nM),^[^ ^19^ ^]^ an endogenous anticoagulant protein that is homologous to PZ. The best fit was obtained with the heterogenous ligand model, which revealed a second binding event with much lower heme‐binding affinity (*K*_D2_ of 8.57 µM), likely due to additional unspecific binding. The fast association (*k*_a1_ of 4.73 × 10^4 ^M^−1 ^s^−1^) and dissociation (*k*_d1_ of 5.83 × 10^−3 ^s^−1^) rates account for a transient nature of the complex formation. Similar kinetic rates were observed in case of other transiently heme‐binding proteins, such as APC.^[^ ^19^ ^]^ For the second binding event a slower association but similar dissociation (*k*_a2_ of 8.45 × 10^2 ^M^−1 ^s^−1^; *k*_d2_ of 7.24 × 10^−3 ^s^−1^) was observed. The UV/vis spectroscopic titration indeed supported heme binding to PZ as evident by the Soret band shift to ≈404 nm. This has been described for other heme‐binding proteins, such as methemalbumin, before and has been attributed to the formation of a more loosely (transiently) bound ferric heme–protein complex.^[^ ^34^ ^,^ ^35^ ^]^ In addition, the titration demonstrated that PZ has only the capacity for binding one heme molecule specifically (Figure 1B,C). Interestingly, this is again very similar to APC, which has been reported to bind one to two heme molecules.^[^ ^19^ ^]^ In contrast to heme, zinc protoporphyrin IX and protoporphyrin IX did not bind to PZ (Figure S1, Supporting Information), demonstrating the specificity of heme binding to PZ and the necessity for the presence of the iron ion for the PZ–heme interaction.

### 2.2 PZ Has One Major HBM in the Peptidase‐Like Domain

**Figure 2:**
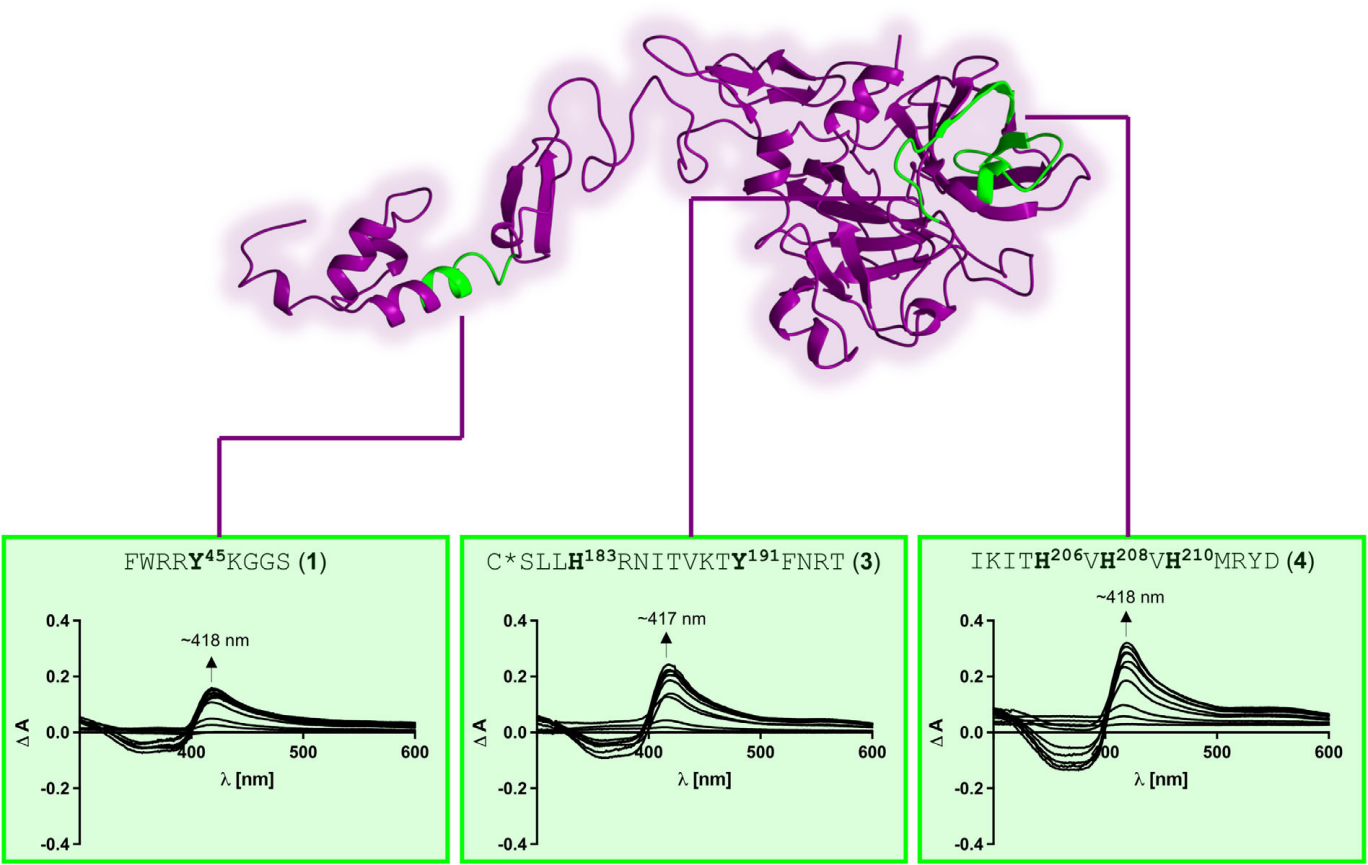
HBMs on the surface of PZ. Synthesized as PZ‐derived peptides, the heme binding affinity of these motifs were determined by UV/vis spectroscopy. HBMs **1** (FWRR**Y** ^**45**^KGGS), **3**, and **4** turned out as potential heme‐binding sites in PZ. All three HBMs showed a high heme‐binding affinity as peptides with *K*_D_ values of 1.04 ± 0.36 µM (**1**), 1.13 ± 0.50 µM (**3**), and 0.79 ± 0.36 µM (**4**). While HBM **1** is found in the Gla domain, HBMs **3** and **4** are located in the S1 peptidase‐like domain of PZ. In motif **3**, the cysteine (*) residue is involved in a disulfide bond. To mimic the blockage for heme binding, a methylated cysteine residue was incorporated in the respective peptide.

Since peptides were demonstrated as suitable models for mapping heme‐binding motifs (HBMs) in proteins,^[^ ^18^ ^,^ ^19^ ^,^ ^36^ ^,^ ^37^ ^]^ herein five PZ‐derived peptides (peptides **1** **–5**) were synthesized (Table S1, Supporting Information), based on a screening of the primary sequence of PZ with HeMoQuest^[^ ^38^ ^]^ and manual refinement. These potential HBMs encompass FWRR**Y** ^**45**^KGGS (**1**), AKNEC(Me)**H** ^**90**^PERT (**2**), C(Me)SLL**H** ^**183**^RNITVKT**Y** ^**191**^FNRT (**3**), IKIT**H** ^**206**^V**H** ^**208**^V**H** ^**210**^MRYD (**4**), and VTRE**H** ^**321**^RGSW (**5**). On peptide level, three from the predicted five HBMs (i.e., **1**, **3**, and **4**) bound heme (**Figure** **2**). Peptides **2** and **5**, motifs that possess only one central histidine residue, did not bind heme, which mainly might be caused by electrostatic repulsion through the acidic glutamic acid residues in both motifs (Figure S2, Supporting Information). In contrast, peptides **1**, **3**, and **4** showed high heme‐binding affinities with *K*_D_ values of 1.04 ± 0.36 µM, 1.13 ± 0.50 µM, and 0.79 ± 0.36 µM, respectively (Figure 2).

Motif **1** has only a tyrosine as coordinating residue but heme binding is stabilized through *p–* *π* stacking effects between the porphyrin ring of heme and the aromatic phenylalanine and tryptophane residues as well as electrostatic interactions between the propionic groups of heme and the positively charged arginine and lysine side chains. However, although suitable for heme binding on peptide level, on protein level no docking pose with a distance below 3 Å between the oxygen atom of tyrosine and the iron ion of heme was found. Same applied for H^183^ and Y^191^ in motif **3**, excluding motifs **1** and **3** as HBMs in PZ. In both cases, loops hindered heme coordination by the respective sites (Figure S3, Supporting Information). In contrast, for motif **4** (HXHXH), a suitable docking pose was identified with a distance of 2.18 Å between the deprotonated nitrogen atom of the central H^208^ and the heme iron ion. During the entire 100 ns, molecular dynamics (MD) simulation heme stayed closely bound to motif **4** with an average distance of 2.18 Å. The Poisson–Boltzmann binding energy estimated to quantify the heme‐motif association was −266.42 kJ mol^−1^. These observations provide corroborative support for the experimental results obtained at peptide level. On protein level, this motif is located in the peptidase S1‐like domain of PZ, which has no catalytic activity in PZ due to mutations in the active site. However, in earlier reports this domain was proposed to participate in the interaction of PZ with thrombin,^[^ ^28^ ^]^ which could be influenced in the presence of heme.

**Figure 3:**
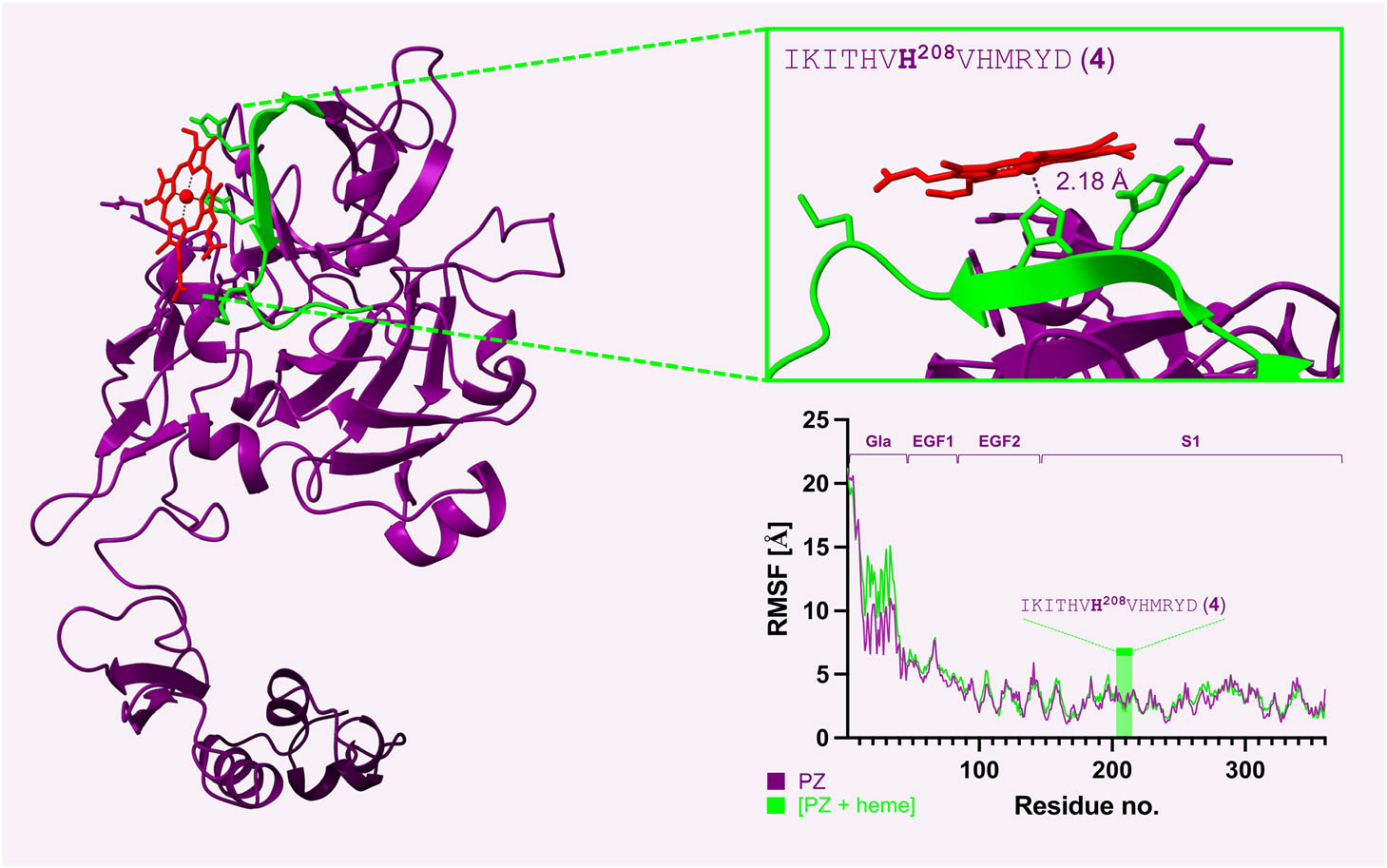
In silico studies confirm heme binding to HBM **4** on protein level. Molecular docking was only successful for HBM **4** with a distance of 2.18 Å between the deprotonated nitrogen of H^208^ and the heme iron ion. During a 100 ns long MD simulation, heme stayed bound to H208. The RMSF plot demonstrates that the protein gained higher flexibility in the Gla domain when heme was bound to HBM **4**. Gla, Gla domain; EGF1, EGF 1‐like domain; EGF2, EGF 2‐like domain; S1, Peptidase S1‐like domain.

HBM **4**, especially the coordinating residue H^208^, is highly conserved among different organisms, suggesting not only the potential for heme binding to the protein in other organisms but also the importance of the histidine residue (Table S2, Supporting Information). In contrast, H^208^ and the surrounding motif is not conserved in the related vitamin K‐dependent proteins APC, thrombin, factor VIIa, factor IXa, FX, and protein S (Table S3, Supporting Information). Although showing ≈15–40% identity (highest with APC) and ≈35–70% (highest with APC and thrombin) similarity, the histidine residue essential for heme binding is not found in the respective sequences of the related proteins. The highly similar APC was shown to bind heme before via the motifs WIHG**H** ^**391**^IRDK and TGWG**Y** ^**289**^HSSR.^[^ ^19^ ^]^ These motifs were not conserved in the sequence of PZ at all, again supporting differences in the heme‐binding sites of the two proteins (Figure S4, Table S4, Supporting Information). As indicated by increased RMSF, heme bound to HBM **4** induced a higher flexibility in the N‐terminal Gla domain of the protein (**Figure** **3**), suggesting heme‐induced conformational and dynamic changes in the protein. Interestingly, heme affected the dynamics of the Gla domain in APC as well but there it conferred a loss of flexibility.^[^ ^19^ ^]^ Similar as in PZ, heme increased the flexibility of certain regions in FVIII as well, thus hampering its function as a cofactor.^[^ ^18^ ^]^ As in all vitamin K‐dependent proteins, the Gla domain in PZ is crucial for the coordination of calcium ions to its characteristic γ‐carboxy glutamic acid residues, which, in turn, facilitates the association with the negatively charged phospholipid membrane of activated platelets. This enables anchoring and positioning of ZPI to the membrane, where it can inhibit factor Xa.^[^ ^22^ ^]^ The changes in structural dynamics of the Gla domain could potentially affect this function of the protein.

### 2.3 Heme Influences the Function of PZ

**Figure 4:**
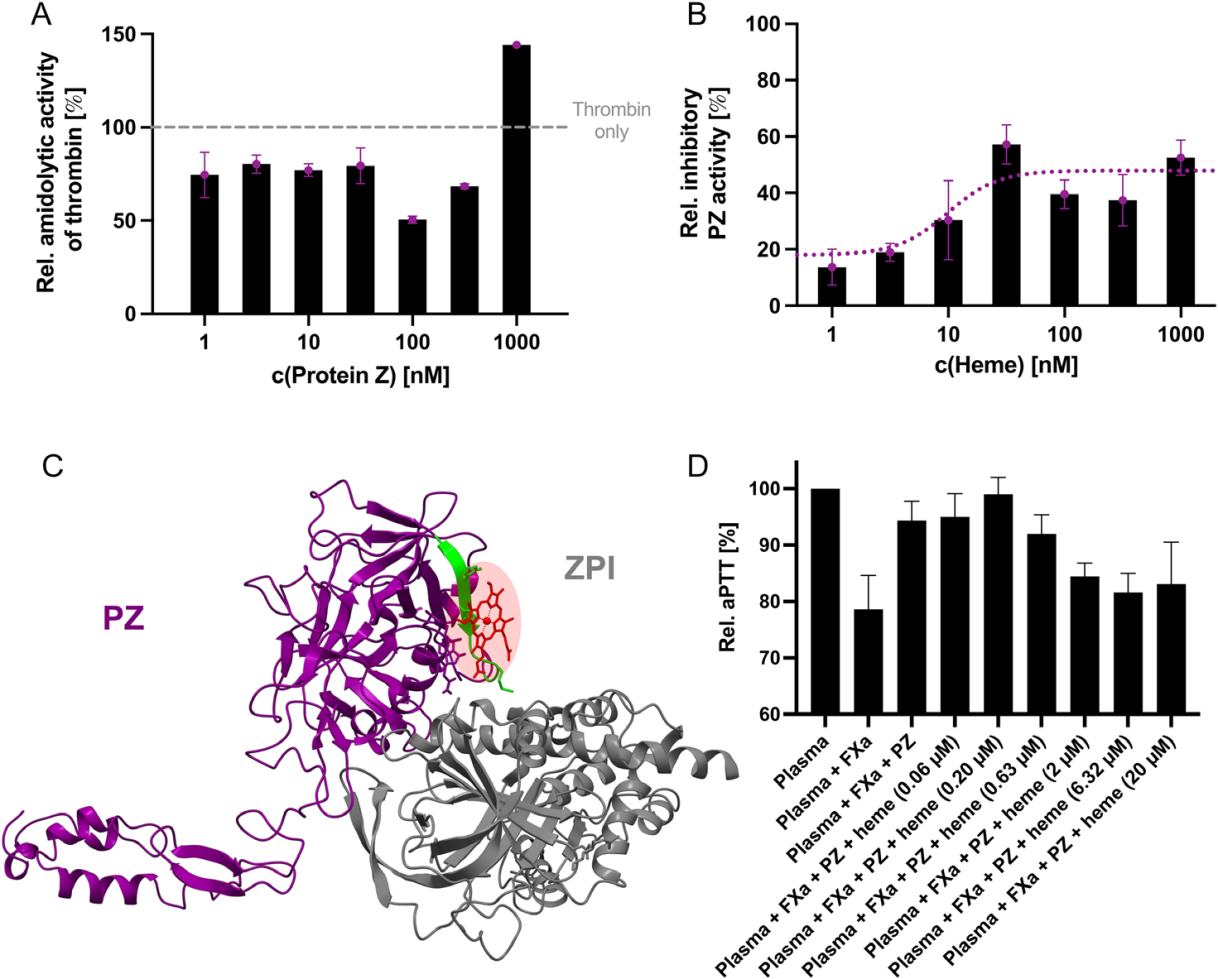
Insights into the impact of heme binding on the function of PZ. A) Effect of PZ (11000 nM) on the amidolytic activity of thrombin (30 nM) tested in a previously established chromogenic assay.^[^ ^19^ ^,^ ^28^ ^]^ In the range of PZ:thrombin ratios of 1:30 to ≈10:1, PZ reduced the amidolytic activity of thrombin to a remaining level of ≈5080%. However, at the highest PZ concentration applied (1 µM), the amidolytic activity of thrombin was increased to ≈140%, turning the functional effect of PZ in an additional functional direction that has not yet been identified by others before. B) The inhibitory activity of PZ (10 nM) against thrombin (30 nM) is slightly increased in the presence of heme in a concentration‐dependent manner, characterized by an EC_50_ of 12.70 ± 6.36 nM. C) Structure of the PZ–ZPI complex (derived from PDB 3H5C^[^ ^42^ ^]^). with heme bound to H^208^ in PZ. Although the heme‐binding site (HBM 4) is not directly located in the interface of PZ and ZPI, it is very close to it, suggesting that this interaction might be affected by allosteric effects as well. D) The anticoagulant function of PZ (32 nM) against FXa (0.1 nM) is inhibited by heme (0.06–20 µM) in a concentration‐dependent manner, characterized by an IC_50_ of 0.83 ± 0.21 µM. Thereby, heme is able to restore the procoagulant activity of FXa. These results demonstrate that the anticoagulant function of the PZ–ZPI complex is abolished by heme.

To analyze the effect of heme on PZ, first an earlier proposed inhibitory effect of PZ on thrombin's amidolytic activity was analyzed in the presence of heme.^[^ ^28^ ^]^ While testing for the optimal PZ concentration, a slight inhibitory effect (≈20%) toward the amidolytic activity could be confirmed in case of the PZ:thrombin ratios of 1:15 to 21:1, as has been described earlier as well (**Figure** **4**A).^[^ ^28^ ^]^ This effect has been attributed to either PZ binding to the active site of thrombin and, thus, steric hindering of substrate binding, or the induction of conformational changes in thrombin by PZ.^[^ ^28^ ^]^ For the first time, also an increased activity of thrombin could be detected at the highest PZ concentration applied (1 µM) and a ratio of ≈30:1 PZ:thrombin (Figure 4A). Hogg and Stenflo (1991) also observed the ≈20% inhibition of the activity of thrombin in the presence of PZ; however, they only tested PZ:thrombin ratios of ≈1:9 to ≈5:1,^[^ ^28^ ^]^ meaning a less broad concentration range than reported herein. Thus, this is the first report on an increase of the amidolytic activity of thrombin in the presence of a 30‐fold excess of PZ. This might support the reported paradox role of PZ a procoagulant and anticoagulant protein in the physiological context as well.^[^ ^39^, ^40^, ^41^ ^]^

For the analysis of a functional impact of heme on PZ, herein the focus was first laid on the thrombin activity‐dampening effect of PZ. This assay was also performed with some controls (Figure S5, Supporting Information), including thrombin only in the presence of heme and substrate. Unexpectedly, heme reduced the amidolytic activity of thrombin at the highest concentration applied as well (Figure S6, Supporting Information). In the lower area (<1000 nM), heme did not significantly influence the activity of thrombin (Figure S6, Supporting Information). Though the effect occurs only at the highest concentrations and seems thus relatively unspectacular and negligible in comparison to the effect of heme on other coagulation proteins, SPR analysis revealed for the first time direct heme binding to thrombin. With a *K*_D1_ of 92.9 nM and *K*_D2_ of 709 nM, the heme‐binding affinity of thrombin is slightly higher but still in the same range as of APC and PZ (Figure S7, Supporting Information). Since the effect of heme on the amidolytic activity of thrombin is rather low, the effect of heme on other functions of thrombin, such as in inflammation, should be analyzed in future. In addition, thrombin might contribute to the heme‐binding capacity of plasma which might support the heme‐scavenging system in most severe hemolytic conditions.

While PZ showed an inhibitory effect of 20%, this was increased in the presence of heme in a concentration‐dependent fashion up to 52.58 ± 6.28% with an EC_50_ of 12.70 ± 6.36 nM (Figure 4B). Considering the preincubation of 30 min with PZ and heme only, it is highly probable that the effect observed mainly originates from the PZ–heme interaction and the observed effect on thrombin occurs only in the absence of PZ. Both effects tend toward a reduced thrombin activity, which would mean in a physiological context that blood clotting would be impaired. However, this function of PZ has only been demonstrated in vitro and has not been confirmed in the physiological context so far. Thus, these results only suggest that heme might have an effect on, e.g., the dynamics of the protein, which would be in line with the in silico results obtained herein.

The effect of heme on the major role of PZ as a cofactor of ZPI as a matter of protein–protein interactions could not be directly tested due to the fact that plasma‐derived, mature ZPI could not be obtained. However, to at least estimate a potential influence of heme on the major role of PZ the successfully simulated PZ–heme complex was overlayed with the available crystal structure of the PZ–ZPI complex (PDB: 3H5C^[^ ^42^ ^]^). Notably, the heme‐binding site in PZ is rather close by the interface of PZ and ZPI (Figure 4C), suggesting a potential regulating impact on this interaction by heme. To prove this, a modified clotting assay with focus on the effect of the inhibitory cofactor function of PZ in the presence of heme was performed. In this setup, PZ was able to block the procoagulant effect of FXa completely. However, in the presence of higher heme levels (≥2 µM), the procoagulant function of FXa was restored, demonstrating the inhibition of the anticoagulant cofactor function of PZ in the presence of heme (IC_50_ of 0.83 ± 0.21 µM; Figure S8, Supporting Information). In contrast, heme, PZ and the PZ–heme complex alone did not exert any significant impact on the procoagulant function of FXa or on clotting of pure plasma (Figure S9, Supporting Information).

**Figure 5:**
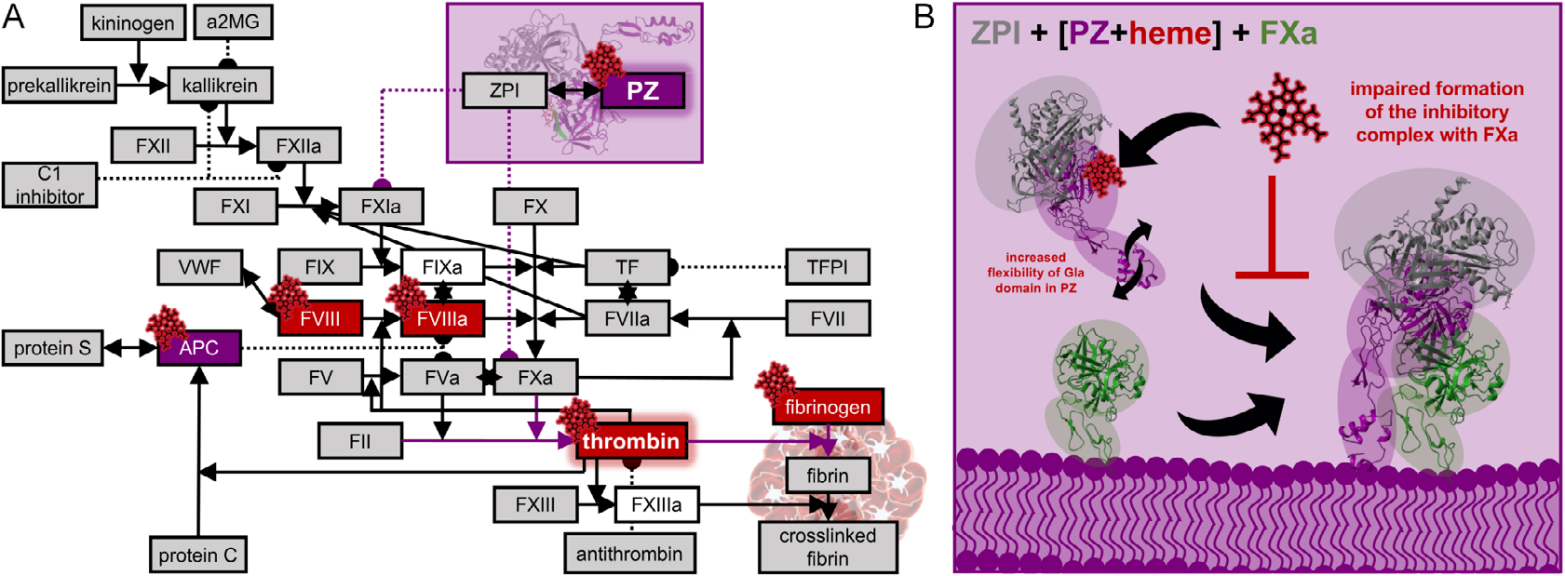
Physiological role of PZ in the coagulation cascade and the potential pathophysiological effect of heme on PZ. A) The coagulation cascade and anticoagulant systems with so far known heme‐binding proteins, including those characterized in this study, are depicted. Previously, there was no report on PZ as a heme‐binding protein. In this study, heme binding to PZ was characterized with a *K*_D_ value in the nanomolar range. Procoagulant factors known to bind heme (i.e., FVIII/FVIIIa,^[^ ^17^ ^,^ ^18^ ^]^ fibrinogen,^[^ ^15^ ^,^ ^16^ ^]^ thrombin) are highlighted in red. Anticoagulant proteins known to bind heme (i.e., APC^[^ ^19^ ^]^ and PZ) are highlighted in purple. Proteins that were previously demonstrated to not bind heme^[^ ^19^ ^]^ or were not yet analyzed for their heme‐binding behavior are shown in white and gray boxes, respectively. B) As demonstrated previously for other proteins as well, heme affects the function of PZ upon direct binding to the protein. In a one‐stage plasma‐based clotting assay, heme was capable of reducing the anticoagulant cofactor actions of PZ against FXa. Under hemolytic conditions, this would account for a support of prothrombotic side effects through elimination of an anticoagulant system in the intravascular compartment. The impairment might occur due to an impact of heme on the dynamics of the protein, especially concerning the Gla domain. Since this domain is particularly important for the interaction of PZ with FXa and thus for the recruitment and proper orientation of ZPI toward FXa, this step in the formation of the inhibitory protein complex might be disturbed in the presence of heme.

The observed impairment of the anticoagulant role of PZ could result from an influence of heme on the protein's dynamics, particularly affecting the Gla domain, as also observed in the MD simulation studies. As this domain plays a crucial role in the interaction of PZ with FXa and thereby in the recruitment and correct orientation of ZPI toward FXa, the presence of heme may disrupt this step in the formation of the inhibitory protein complex (**Figure** **5**).

## 3 Conclusion

In a screening for novel, so far unknown heme‐binding proteins within the endogenous blood coagulation inhibitor system, PZ came up as a suitable candidate due to its homology with APC, which has been earlier identified as the first heme‐binding anticoagulant protein. Indeed, herein, binding of one heme molecule to the surface of PZ was demonstrated by means of SPR spectroscopy, UV/vis spectroscopy and in silico approaches. Similar to APC, PZ bound heme with high affinity in the nanomolar range. Furthermore, the use of PZ‐derived peptides allowed for the experimental approval of putative heme‐binding sites at the surface of PZ, revealing FWRR**Y** ^**45**^KGGS (**1**), C(Me)SLL**H** ^**183**^RNITVKT**Y** ^**191**^FNRT (**3**), and IKIT**H** ^**206**^V**H** ^**208**^V**H** ^**210**^MRYD (**4**) as suitable motifs. Molecular docking and MD simulation studies demonstrated, however, that heme binding was only possible to H^208^ in HBM **4** due to steric hindrance by loops close to the other motifs. In addition, the *in silico* studies suggested an increased flexibility of the Gla domain of PZ in the presence, suggesting that essential interactions with, for example, FXa might be influenced. A potential functional consequence of heme binding to PZ was explored by a chromogenic and a clotting assay, demonstrating that heme influences the function of PZ. In particular, the anticoagulant properties are dampened by heme, suggesting that heme has an impact on the inhibitory complex formation of PZ‐ZPI with FXa (Figure 5).

Finally, this study uncovered two further, so far unknown protein from the blood coagulation system as heme‐binding proteins, namely, PZ and thrombin. Interestingly, this study suggests that PZ lines up to the proteins that support a prothrombotic tendency in the presence of heme. As in case of APC, a heme‐induced inhibition of the anticoagulant function of the protein might lead to an acceleration of the known prothrombotic effects of heme under hemolytic conditions. In future, further studies are necessary to not only understand the (patho)physiological role of PZ in high heme conditions but also the overall regulating effects occurring through heme–protein interactions in the blood coagulation system in hemolysis‐induced coagulation disorders.

## 4 Experimental Section

4.1

4.1.1

##### Reagents

Hemin, herein referred to as “heme,” was purchased from Merck KGaA. The stock solution of heme (1 mM) was prepared in 30 mM NaOH as described previously.^[^ ^19^ ^]^ For the experiments, the heme stock solution was then diluted in the desired buffer. Human protein Z (HCPZ‐0220), human α‐thrombin (HCT‐0020), and human factor Xa (HCXA‐0060) were obtained from CellSystems GmbH. The chromogenic substrate S‐2238 was bought from Haemochrom Diagnostica GmbH. Dade Actin FSL and citrated pooled plasma were obtained from Siemens Healthineers and Biowest, respectively. Amino acid derivatives and coupling reagents were obtained from Iris Biotech GmbH. Solvents and salts were purchased from VWR International GmbH or Carl Roth GmbH. Amine coupling reagents and the CM5 series S sensor chip were acquired from Cytiva Germany GmbH. Trifluoroacetic acid for peptide cleavage was kindly provided by Solvay GmbH.

##### SPR Binding Studies

SPR experiments were conducted on a Biacore 8K instrument (Cytiva) to investigate the interaction of PZ with heme, as established before.^[^ ^19^ ^,^ ^35^ ^,^ ^37^ ^,^ ^43^ ^]^ In brief, PZ (10 µg mL^−1^) was dissolved in 10 mM sodium acetate buffer (pH 3.5) and immobilized onto a CM5 sensor chip via standard amine coupling, resulting in a final immobilization level of 767 RU. Heme (stock solution in 30 mM NaOH) was further diluted in running buffer (10 mM HEPES, 150 mM NaCl, and 0.05% Tween 20, pH 7.4). The same was performed with zinc (II) protoporphyrin IX and protoporphyrin IX (Frontier Scientific). Binding kinetics were assessed by injecting seven increasing concentrations of heme (187.5–12,000 nM) in a single‐cycle kinetics format at 25 °C and a flow rate of 30 µL min^−1^. Data analysis was performed by global fitting using Biacore Insight Evaluation Software, version 3.0.12.15655 (Cytiva).

##### UV/vis‐Spectroscopic Titration of Heme to PZ

As described earlier for other proteins,^[^ ^18^ ^,^ ^35^ ^]^ stepwise titration of heme to PZ was carried out. In each step, 5 μL of a 90 μM heme solution was added to 45 μL of PZ (initial concentration: 10 μM). The titration was performed for five steps in total, aiming for the analysis of heme binding to PZ up to a ratio of 1:5 (PZ:heme). After each addition, the mixture was incubated for 30 min at room temperature, followed by measurement of the absorbance spectrum from 300 to 600 nm using an Infinite 200 Pro spectrophotometer (Tecan). As a control, the same titration was performed with 1× PBS (pH 7.4) instead of PZ. To evaluate binding, difference spectra were calculated by subtracting the spectra of heme only from those of the heme–PZ mixtures. Additionally, the absorbance differences at the wavelength of absorbance maximum (i.e., 404 nm) were plotted against the PZ:heme molar ratio to estimate the number of heme‐binding sites in PZ.

##### Prediction of Heme‐Binding Sites in PZ

Heme‐binding sites in PZ were predicted by using the webserver HeMoQuest (http://131.220.139.55/SeqDHBM/)^[^ ^36^ ^,^ ^38^ ^]^ for screening of potential HBMs starting from the primary sequence of mature PZ. In total, 26 potential cysteine‐, histidine‐, and tyrosine‐based HBMs were shortlisted as nine amino acid long sequences. These were manually evaluated with respect to their accessibility for heme binding in the available crystal structures of PZ (PDB: 3F1S^[^ ^23^ ^]^ and 3H5C^[^ ^42^ ^]^). Seventeen motifs (around C^51^, C^56^, C^62^, C^71^, C^73^, C^89^, C^97^, C^101^, C^112^, C^125^, C^131^, C^163^, C^179^, C^233^, C^241^, and C^301^) were withdrawn due to their involvement in disulfide bonds and the resulting steric blockage of the cysteine residues. In addition, the two suggested HBMs around Y340 and Y391 were excluded since there were buried and not available on the surface of the protein and, thus, not accessible for transient heme binding. The remaining 10 HBMs were partially overlapping and, thus, combined, yielding in a final selection of five potential heme‐binding sites [i.e., around Y^45^ (**1**), H^90^ (**2**), H^183^/Y^191^ (**3**), H^206^/H^208^/H^210^ (**4**), and H^321^ (**5**)] on the surface of PZ.

##### Synthesis and Analytical Characterization of PZ‐Derived Peptides 1–5

The predicted HBMs of PZ were synthesized as PZ‐derived peptides, as described before for other proteins as well. In brief, the peptides were synthesized as 9mer to 17mer peptides **1** **–5** by solid‐phase peptide synthesis using standard Fmoc strategy. In case of HBMs that contained a cysteine residue (**2** and **3**), the cysteine was incorporated as methylated cysteine to mimic the steric hindrance for heme binding caused by their involvement in disulfide bonds on protein level. After cleavage from resin, peptides were purified by preparative RP‐HPLC (ECS12, ECOM) equipped with C18 column (250 × 32 mm, 5 μm particle size, 100 Å pore size). The purified peptides were analytically characterized by analytical RP‐HPLC equipped with a Nucleodur 300‐5 RP column (C18, 250 × 4.6 mm, 5 μm particle size, 300 Å pore size), MALDI‐TOF mass spectrometry (UltrafleXtreme, Bruker Daltonics GmbH & Co. KG), and thin‐layer chromatography (Table S1, Supporting Information).

##### UV/vis Spectroscopic Interaction Studies of PZ‐Derived Peptides with Heme

Heme binding to PZ‐derived peptides **1** **–5** was analyzed by UV/vis spectroscopy, as previously reported for other protein‐derived peptides as well.^[^ ^18^ ^,^ ^19^ ^,^ ^35^ ^,^ ^43^ ^,^ ^44^ ^]^ In brief, each peptide (10 µM) was incubated for 30 min with heme in 100 mM HEPES buffer (pH 7.0) for 30 min. Absorbance spectra were recorded in the range of 300–600 nm on a Infinite 200 Pro spectrophotometer. Difference spectra were generated by subtraction of the single absorbance data of heme and the peptide, respectively, from the data of the heme–peptide complex. Difference spectra were plotted with GraphPad Prism, version 10.5.0. *K*_D_ values were calculated using the equation from Pîrnău and Bogdan (2008).^[^ ^45^ ^]^

##### Molecular Docking and MD Simulations

Only two incomplete crystal structures of PZ in complex with ZPI are available (PDB: 3F1S^[^ ^23^ ^]^ and 3H5C^[^ ^42^ ^]^). Therein, several parts of PZ are missing (A^1^–K^86^, E^139^–Q^146^, T^195^, G^267^–S^273^ in PDB 3F1S^[^ ^23^ ^]^ and A^1^–G^48^ in PDB 3H5C^[^ ^42^ ^]^). Thus, the first step was the adjustment of the available AlphaFold^[^ ^46^ ^]^ structure of PZ (AF‐P22891‐F1), encompassing the manual insertion of known disulfide bonds as well as the removal of the signal peptide and the propeptide (40 residues at the N‐terminus). This structure of PZ was energy minimized using the steepest descent protocol followed by a simulated annealing approach, both executed with macro md_run in YASARA, version 25.1.13, as described earlier.^[^ ^19^ ^,^ ^47^ ^]^ This optimized structure was then subjected to a 100 ns MD simulation to generate an equilibrated conformational ensemble of PZ (Figure S10, Supporting Information). To explore heme binding to the experimentally validated putative HBMs (**1**, **3**, and **4**), focused ensemble docking with the macro dock_runensemble was performed, as described previously.^[^ ^18^ ^,^ ^19^ ^]^ Clustering of the resulting poses led to list of possible heme–PZ complex conformations, which were then manually analyzed with respect to the distance of the heme iron ion and the potential coordinating residue. Poses were considered as suitable when the distance between the iron ion and the nonprotonated nitrogen atom in histidine or the hydroxyl oxygen in tyrosine was less than 3 Å. The best docked complex was first refined with the macro md_refine and then subjected to MD simulations for 100 ns (md_run; Figure S10, Supporting Information). Analysis of the MD trajectories was performed in YASARA, version 25.1.13. The binding energy was calculated using the Poisson–Boltzmann method implemented in the macro md_analyzebindingenergy in YASARA. Structural graphics and plots were generated in UCSF ChimeraX, version 1.8,^[^ ^48^ ^]^ and GraphPad Prism, version 10.5.0, respectively.

##### Analysis of the Thrombin‐Inhibiting Activity of PZ in the Presence of Heme

The analysis of the function of PZ in the presence of heme is challenging, as PZ is not an active enzyme and a cofactor of ZPI, which is unfortunately only available as fragmented or tagged forms, rendering the direct analysis of PZ–ZPI interaction in the presence of heme impossible currently.

Hogg and Stenflo (1991) demonstrated that human PZ has a slight inhibitory effect toward the amidolytic activity of thrombin.^[^ ^28^ ^]^ Herein, an earlier established chromogenic thrombin amidolytic activity assay system was thus used, inspired by Hogg and Stenflo.^[^ ^19^ ^,^ ^28^ ^]^ PZ (final concentration: 10 nM) was first incubated for 30 min with different heme concentrations (final concentrations: 1–1000 nM). Afterward, the heme–PZ mixture was incubated with thrombin for 10 min and the PZ–thrombin mixture (50 µL; final thrombin concentration: 15 nM) was transferred onto a 96‐well plate (Greiner Bio‐One GmbH). Finally, 5 µL of the substrate solution (H‐D‐Phe‐Pip‐Arg‐pNA x 2 HCl (S‐2238); final concentration: 273 µM) was added. Immediately, kinetic measurements at 405 nm were started, using an Infinite 200 Pro spectrophotometer. Heme only, thrombin only, and PZ‐heme only served as controls. For evaluation, the thrombin activity was determined as the change of absorbance per second and always normalized against the basal thrombin activity without PZ. Data were analyzed as the mean of triplicate and the IC_50_ was calculated by nonlinear regression (GraphPad Prism, version 10.5.0).

##### Investigation of the Procoagulant Activity of FXa in the Presence of the PZ–heme Complex

To estimate an effect on molecular level, the structure of the PZ–ZPI complex (PDB: 3H5C^[^ ^42^ ^]^) was first inspected with heme attached to PZ. The anticoagulant function of PZ was then analyzed in the presence of heme by a one‐stage clotting assay, which was inspired by previous work from us and others.^[^ ^19^ ^,^ ^27^ ^]^ The aPTT‐based assay was performed with the ball coagulometer KC 4 A (Amelung, Lemgo, Germany). PZ (320 nM) was preincubated with different concentrations of heme (0–100 µM) for 30 min in 1x PBS, pH 7.4. Afterward, 50 µL of the PZ–heme mixture was incubated with 50 µL of Actin FSL, 50 µl CaCl_2_ (30 mM), and 50 µL FXa (0.5 nM) at 37 °C for 3 min. Finally, 50 µL citrated pooled plasma was added and the clotting time was automatically recorded. The following mixtures served as controls: 1) 50 µL PBS‐heme + 50 µL Actin FSL + 50 µL CaCl_2_ + 50 µL FXa + 50 µL citrated plasma, 2) 50 µL PBS‐heme + 50 µL Actin FSL + 50 µL CaCl_2_ + 50 µL PBS + 50 µL citrated plasma, 3) 50 µL PZ–heme + 50 µL Actin FSL + 50 µL CaCl_2_ + 50 µL PBS + 50 µL citrated plasma, 4) 50 µL PZ‐PBS + 50 µL Actin FSL + 50 µL CaCl_2_ + 50 µL FXa + 50 µL citrated plasma, 5) 50 µL PZ‐PBS + 50 µL Actin FSL + 50 µL CaCl_2_ + 50 µL PBS + 50 µL citrated plasma, 6) 50 µL PBS + 50 µL Actin FSL + 50 µL CaCl_2_ + 50 µL FXa + 50 µL citrated plasma, and 7) 50 µL PBS + 50 µL Actin FSL + 50 µL CaCl_2_ + 50 µL PBS + 50 µL citrated plasma. Final concentrations in the approach were 6 mM CaCl_2_, 0–20 µM heme, 32 nM and PZ, 0.1 nM FXa. Data were normalized against the clotting time of citrated plasma only (control (7)) and evaluated as the mean of triplicates. The IC_50_ was determined by fitting the curves with nonlinear regression (GraphPad Prism, version 10.5.0).

## Conflict of Interest

The authors declare no conflict of interest.

## Author Contributions

**Marie‐T. Hopp** conceptualized and designed the study. **Marie‐T. Hopp** performed the SPR binding studies and the clotting assay. **Paula Lindemann** performed the remaining experiments and carried out the *in silico* studies. **Paula Lindemann** and **Marie‐T. Hopp** analyzed the data. The manuscript was written by **Marie‐T. Hopp** and finally approved by both authors.

## Supporting information

Supplementary Material

## Data Availability

The data that support the findings of this study are available in the supplementary material of this article.
