# Supplementary material for: Human Protein Z as the Second Known Heme‐Binding Protein from the Endogenous Blood Coagulation Inhibitor System
Source: Chembiochem. 2025 Nov 27;27(1):e202500636. doi: 10.1002/cbic.202500636 (PMC12781162; doi:10.1002/cbic.202500636)
Supplement: Supplementary file 1 — Supplementary Material [file CBIC-27-e202500636-s001.pdf]

Human protein Z as the second known heme-binding protein from the endogenous blood coagulation inhibitor system

Paula Lindemann[a] and Marie-T. Hopp*[a]

[a] P. Lindemann, JProf. Dr. M.-T. Hopp
Bioorganic Chemistry, Chemistry Department
Institute for Integrated Natural Sciences, University of Koblenz
Universitätsstraße 1, 56070 Koblenz (Germany)
E-mail: mhopp@uni-koblenz.de

WILEY VCH

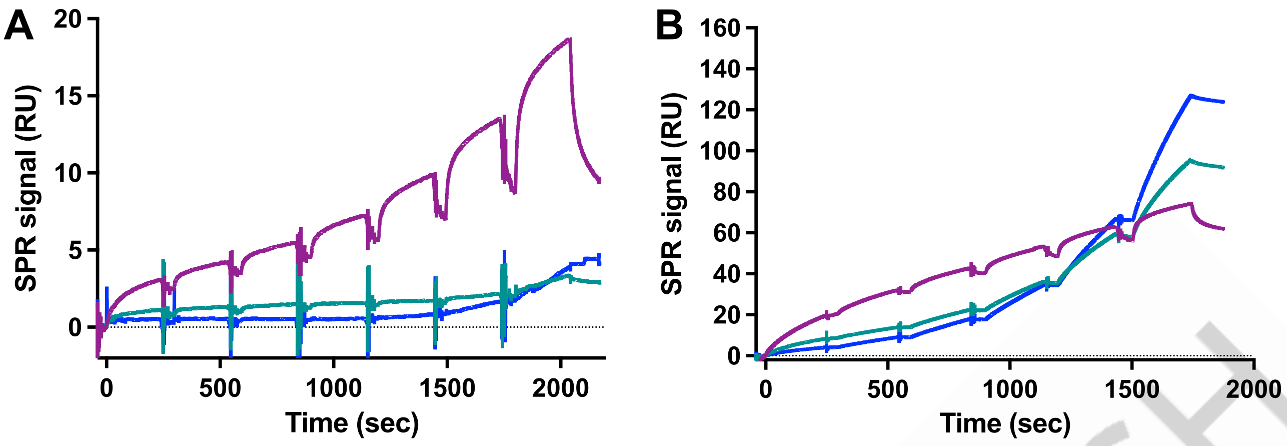

Figure S1. SPR spectroscopic analysis of porphyrin binding to PZ and thrombin. (A) While heme bound to PZ (purple), neither zinc protoporphyrin IX (ZnPPIX; blue) nor protoporphyrin IX (PPIX; green) bound to PZ in the tested concentration range (187.5 nM – 12 µM). (B) In contrast, thrombin showed binding capacity for all applied porphyrins. The ZnPPIX-binding affinity (K_{D1} = 67.2 nM, K_{D2} = 786 nM) is similar to the one for heme, while the affinity for PPIX binding was slightly lower (K_{D1} = 150 nM, K_{D2} = 2.2 µM), demonstrating the importance of the central ion but also less specificity for heme binding than in case of PZ.

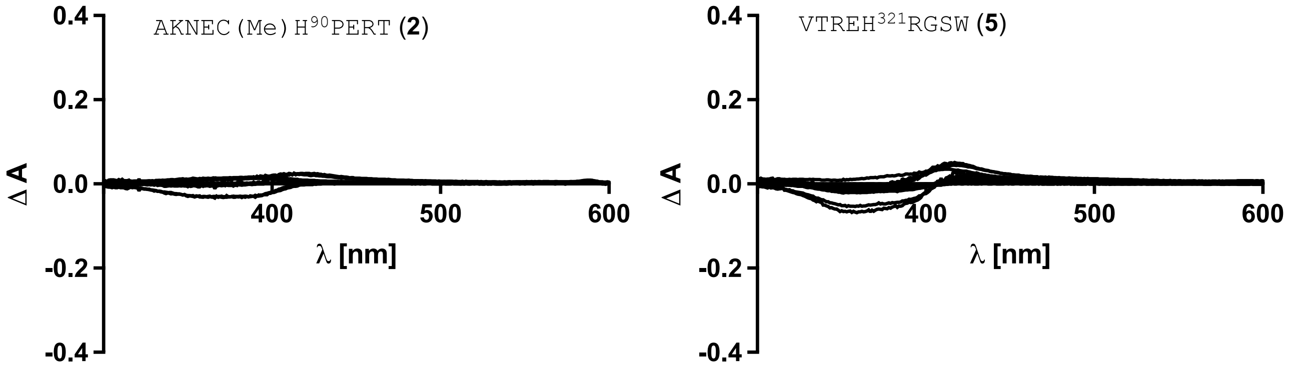

Figure S2. HBMs **2** and **5** did not bind heme as PZ-derived peptides. The difference spectra of the heme-peptide complexes are shown. Each peptide (10 µM) was incubated with different heme concentrations (0.4-40 µM). Difference spectra were generated through subtraction of the heme only and peptide only spectra from the heme-peptide complex spectra. No significant shift of the Soret band of heme could be observed, indicating that heme did not bind to these motifs. Thus, these two motifs are excluded as potential heme-binding sites in PZ.

WILEY-VCH

A FWRR**Y**^{45}KGGS (**1**) **B** CSLL**H**^{183}RNITVKT**Y**^{191}FNRT (**3**)

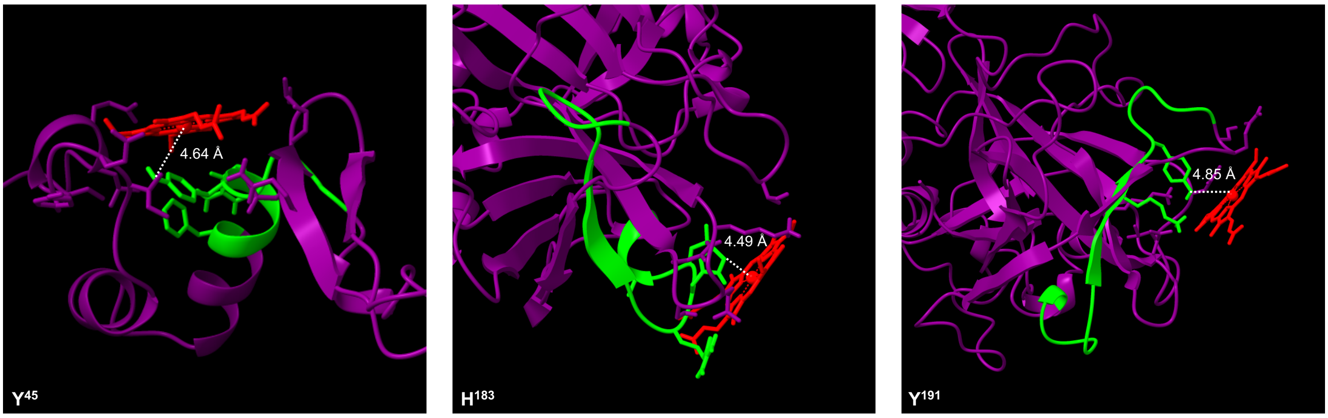

Figure S3. The best docking poses to HBM **1** and **3** showed to large distances (referring to the cutoff of 3 Å) and were does not considered for further evaluation. Notably, in all cases loops around the respective motifs hindered close heme binding to the sites in PZ.

WILEY-VCH

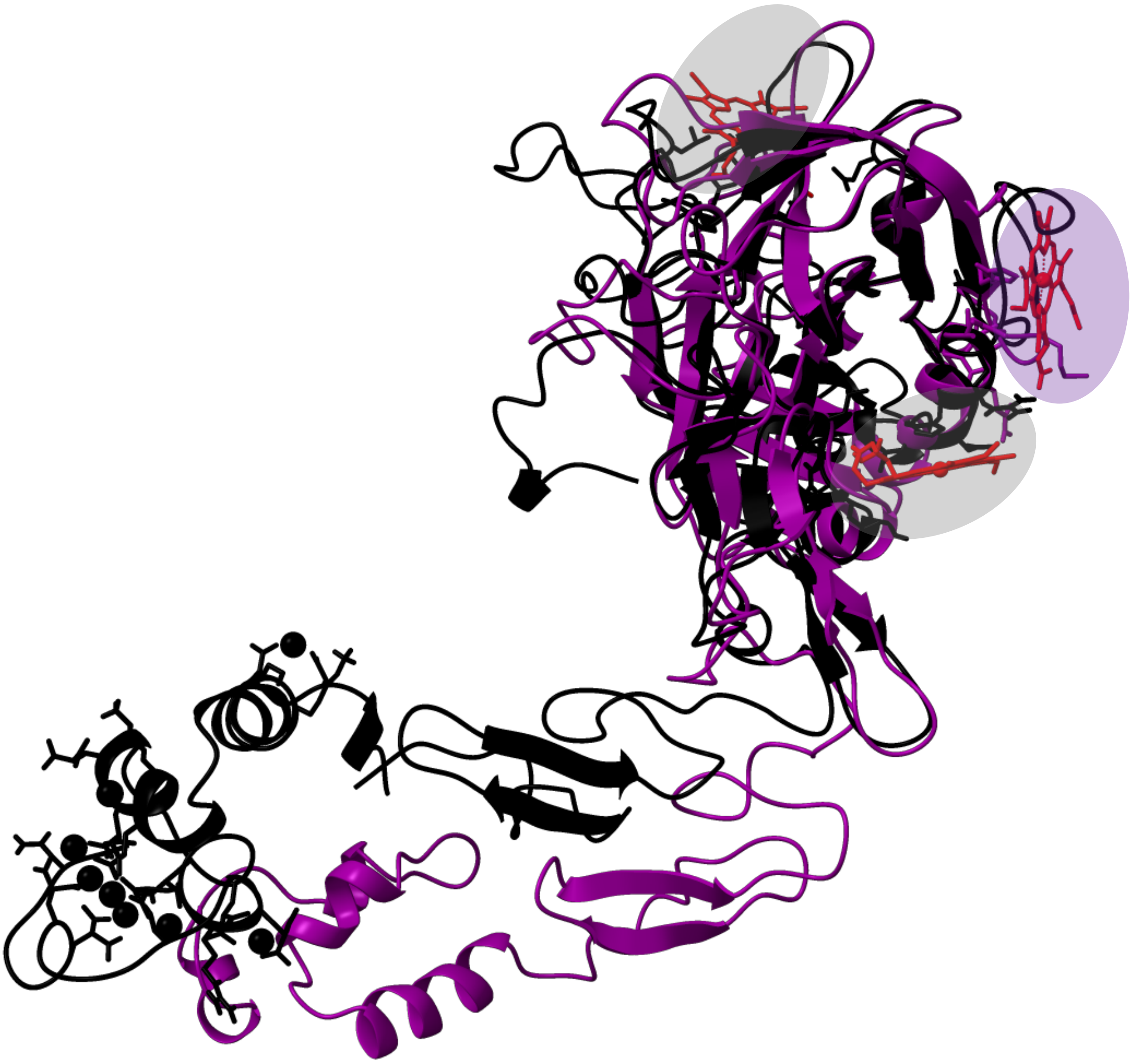

Figure S4. Superimposition of APC (black) with two heme molecules bound to H^{391} (HBM **1a**) and Y^{289} (HBM **2a**), as earlier investigated[1], and PZ (purple) with one heme molecule bound to H^{208}, as found in this study. Although the motifs are not conserved in the respective other protein, HBM 1a (APC) and HBM 4 (PZ) are on the same site of the peptidase S1 domain.

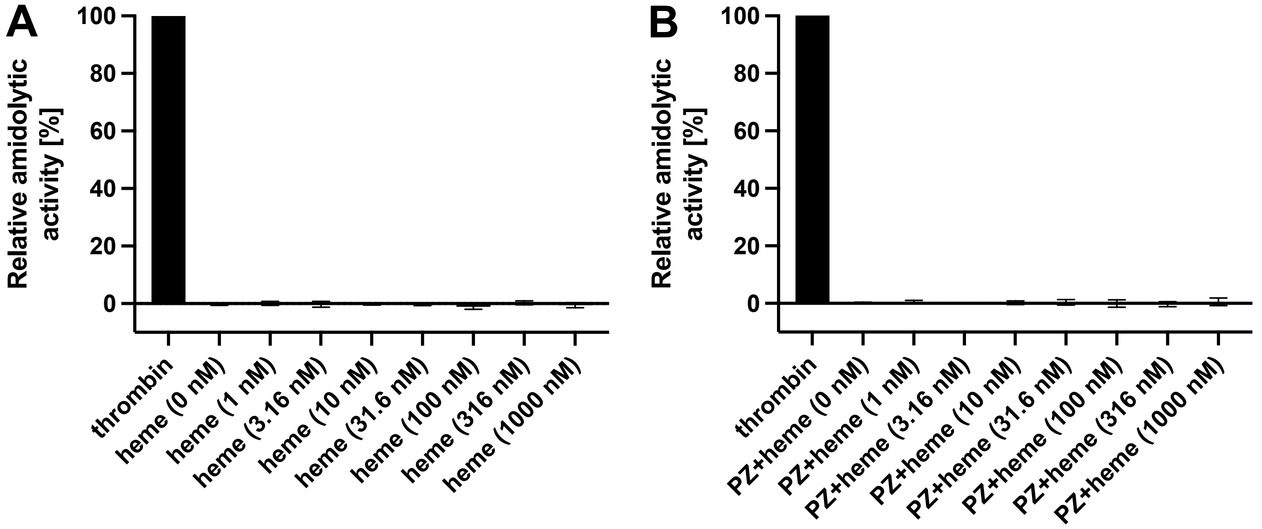

Figure S5. In contrast to the protease thrombin (100 % amidolytic activity), heme (1 – 1000 nM) (A), PZ (10 nM) and the respective PZ-heme complex (B) did not affect the substrate S-2238 (~0 %), which was evident by no changes of the substrate absorbance at 405 nm over time. No substrate conversion could be observed.

WILEY-VCH

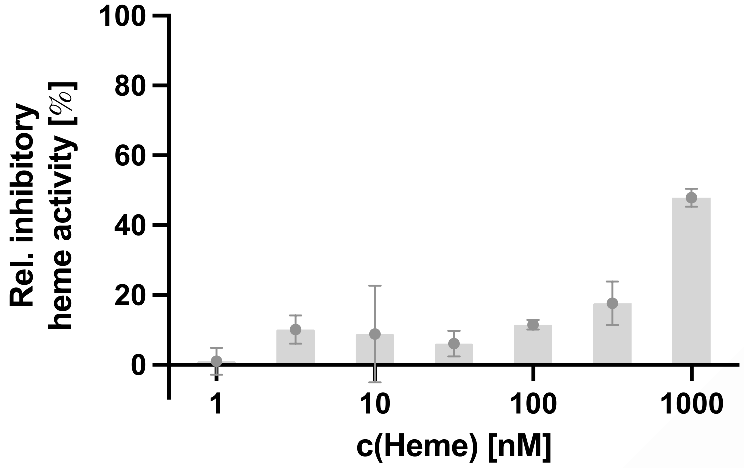

Figure S6. In contrast to an earlier, slightly deviating set-up, heme inhibited the amidolytic activity of thrombin (15 nM) in the highest concentration applied. In this set-up heme was preincubated with thrombin for 10 min before addition of the substrate.

WILEY-VCH

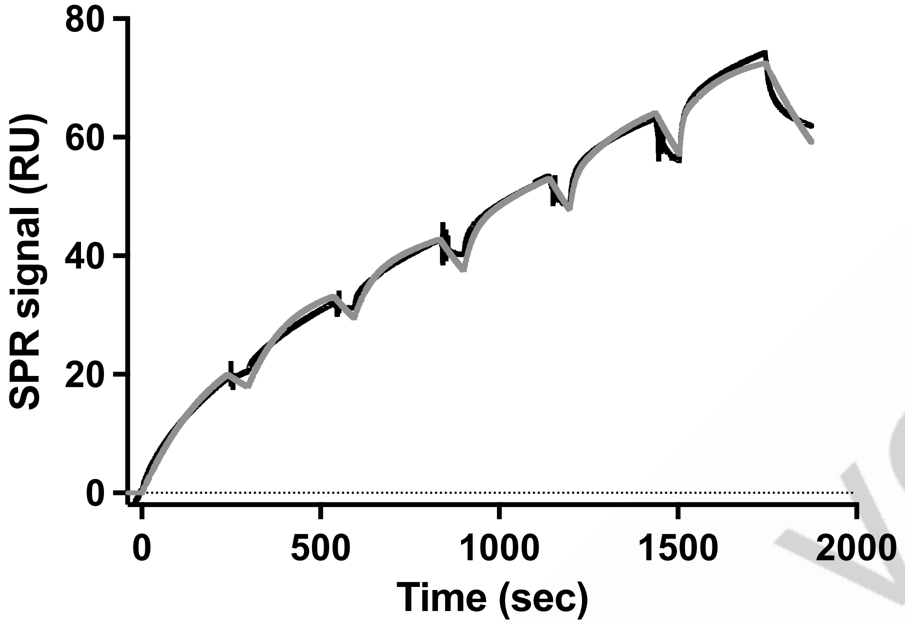

Figure S7. Heme binds to thrombin. For SPR analysis, thrombin (10 µg/ml) was dissolved in 10 mM sodium acetate buffer (pH 6.0) and immobilized onto a CM5 sensor chip via standard amine coupling, resulting in a final immobilization level of 1826.1 RU. Binding kinetics were assessed by injecting six increasing concentrations of heme (187.5 – 6,000 nM) in a single-cycle kinetics format at 25°C and a flow rate of 30 µl/min. According to the best fit, a heterogeneous ligand model was applied, revealing a K_{D1} of 92.9 nM and K_{D2} of 709 nM. In addition, fast association (k_{a1} = 2.89 x 10^4 M^{-1} s^{-1} and k_{a2} = 1.19 x 10^3 M^{-1} s^{-1}) and dissociation (k_{d1} = 2.68 x 10^{-3} s^{-1} and k_{d2} = 8.46 x 10^{-4} s^{-1}) supported the transient nature of heme binding to thrombin.

WILEY-VCH

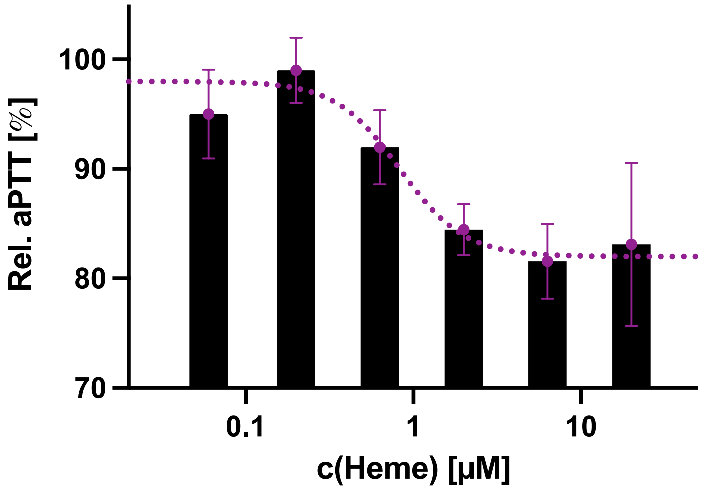

Figure S8. Inhibition of the anticoagulant activity of PZ. The IC50 value was determined as 0.83 ± 0.21 µM.

WILEY-VCH

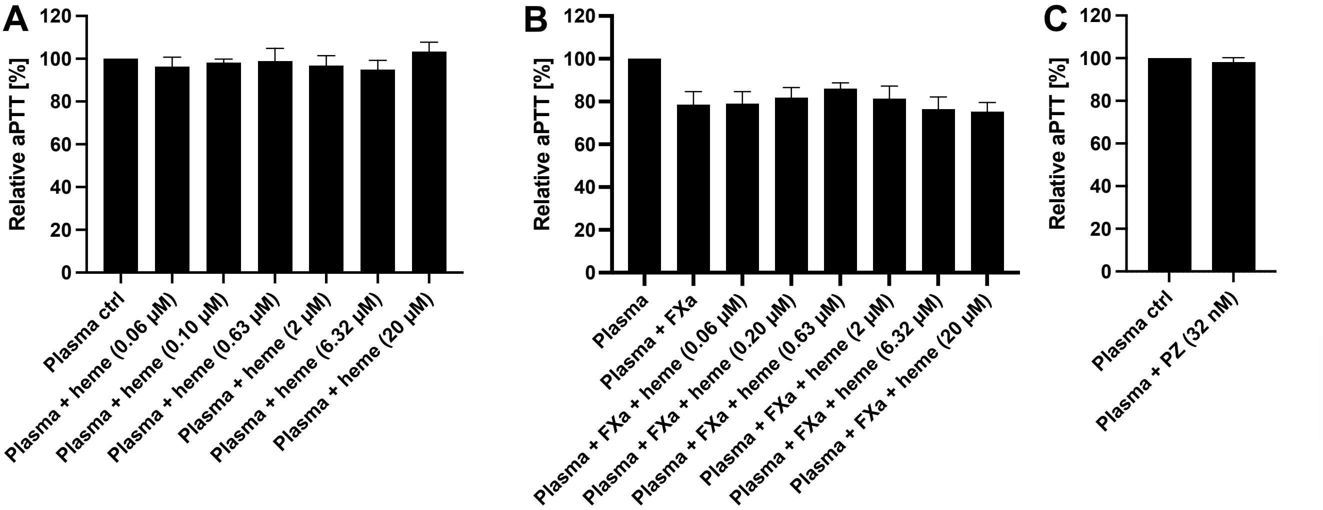

Figure S9. Effekt of heme on the aPTT of plasma (A), effect of heme on the aPTT of plasma in the presence of FXa (B), and the effect of PZ on the aPTT of plasma (C). Heme (final concentrations of 0.06 – 20 µM) did neither affect the clotting time of plasma alone nor the procoagulant effect of FXa (final concentration of 0.1 nM). In the presence of PZ alone the clotting time of plasma was also not significantly influenced.

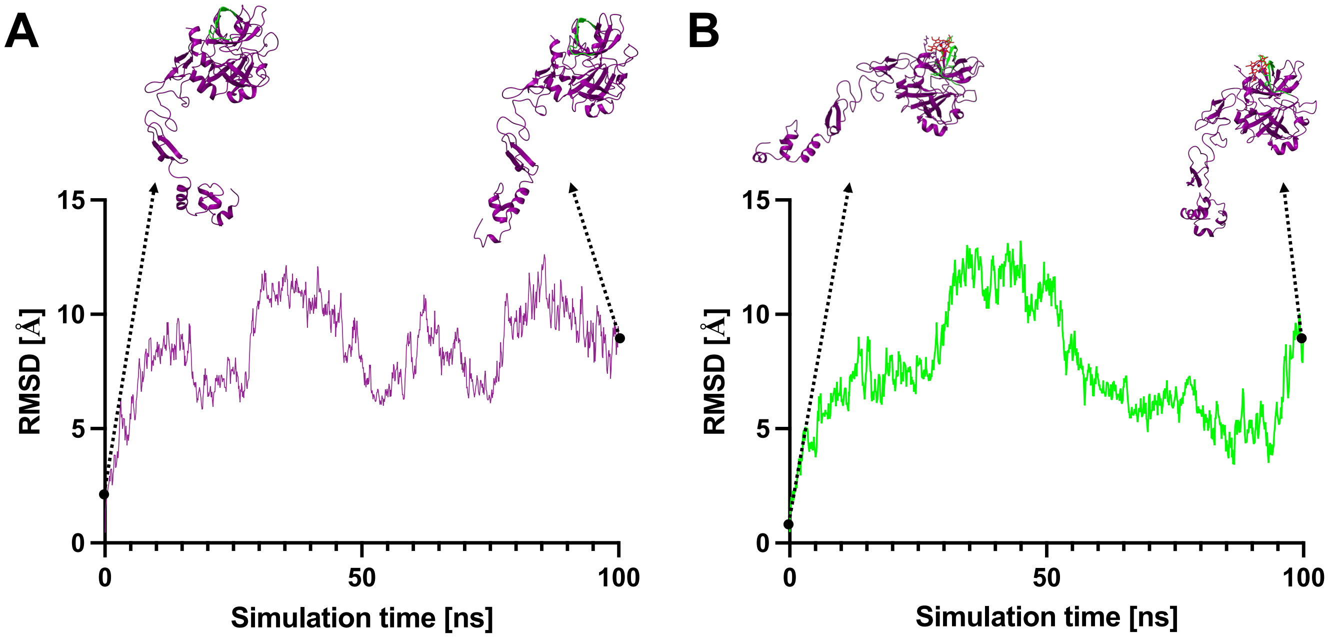

Figure S10. RMSD plot of PZ (A) and the PZ-heme complex (B) during a MD simulation of 100 ns.

WILEY

Table S1. Analytical characterization of protein Z (PZ)-derived peptides **1** - **5**.

		Localization within PZ		Analytical characterization of peptides			Heme binding properties	
**No.**	**Sequence**	**Position[a]**	**Chain**	**M_w (M_w theor.), g/mol[b]**	**HPLC t_R, min**	**TLC R_f**	**K_D, µM[m]**	**Soret shift, nm**
**1**	FWRR**Y**KGGS	41 - 49	Gla/EGF-1	1155.31 (1154.75)	21.31[c]	0.80[g], 0.13[h]	1.04 ± 0.36	~420
**2**	AKNEC(Me) **H**PERT	85 - 94	EGF-2	1197.32 (1196.66)	13.54[d]	0.47[i], 0.89[h]	n.b.	-
**3**	C(Me)SLL**H** RNITVKT**Y**F NRT	179 - 195	S1	2079.43 (2078.33)	14.91[e]	0.83[g], 0.14[j], 0.27[k]	1.13 ± 0.50	~417
**4**	IKIT**H**V**H**V**H** MR**Y**D	202 - 214	S1	1647.95 (1647.04)	15.67[f]	0.16[h], 0.10[j]	0.79 ± 0.36	~418
**5**	VTRE**H**RGSW	317 - 325	S1	1126.23 (1125.74)	18.47[c]	0.13[j], 0.10[l]	n.sat.	~ 421

[a] Positions of motifs are numbered according to the residues within mature protein Z. Potential HRMs were synthesized as nonapeptides, analytical characterized (ESI-MS, HPLC, TLC) and evaluated for their heme-binding by absorbance spectroscopy. [b] Mass peaks were detected as $[M+H]^+$. HPLC conditions were as follows: Water with 0.1 % TFA (eluent A), acetonitrile with 0.1 % TFA (eluent B): [c] 0%-40% eluent B in 40 min, [d] 0%-30% eluent B in 30 min, [e] 15%-45% eluent B in 30 min, and [f] 10%-40% eluent B in 30 min. All peptides were >95 HPLC pure. The following TLC systems were used: [g] 2-butanol/pyridine/water (7:7:6, v/v), [h] acetonitrile (+0.1% TFA)/water (+0.1% TFA) (1:3, v/v), [i] 2-butanol/ammonia (50:2, v/v), [j] acetonitrile (+0.1% TFA)/water (+0.1% TFA) (3:1, v/v), [k] water/acetic acid/methanol (3:2:5, v/v), and [l] pyridine/ethyl acetate/acetic acid/water (5:5:1:3, v/v). [m] K_D values were revealed according to the best fit, suggesting a stoichiometry of 1:1. Gla, Gla-domain; EGF-1, EGF-like 1 domain; EGF-2, EGF-like 2 domain; S1, Peptidase S1 domain.

WILEY-VCH

Table S2. Sequence alignments of the region around HBM **4** in PZ of different organisms.

Organism	Sequence[a],[b]	Identity	Similarity
Homo sapiens (human)	IKITHV**H**^{208}VHMRYD	------------	------------
Bos taurus (bovine)	LHVRGV**H**^{208}VHTRFE	38.46 %	56.25 %
Equus caballus (horse)	IAVQSV**H**^{232}VHMRYE	53.85 %	69.23 %
Cavia porcellus (guinea pig)	ARVRHA**H**^{217}VHMHYD	53.85 %	69.23 %
Rattus norvegicus (rat)	IRIKSA**H**^{228}VHMRYD	69.23 %	76.92 %
Mus musculus (mouse)	IRIKST**H**^{207}VHMRYD	69.23 %	76.92 %
Xenopus laevis (frog)	IMVKMK**H**^{259}PHTGHS	23.08 %	38.46 %

[a] Identical amino acid residues are highlighted in green, similar amino acid residues are displayed in yellow. The original sequence of HBM **4** in human PZ is shown in purple. [b] Central amino acid residues are numbered according to the mature proteins.

WILEY-VCH

Table S3. Sequence alignments of the region around HBM **4** in human PZ with other vitamin K-dependent proteins of the human blood coagulation system.

Proteins	Sequence[a],[b]	Identity	Similarity
Human protein Z (PZ)	IKIT HV**H**208V HMRYD	-----------	-----------
Human activated protein C (APC)	LDIK EVF244V HPNYS	38.46 %	46.15 %
Human thrombin (FIIa)	EKISMLEKIY250I HPRYN	31.25 %	62.50 %
Human factor VIIa (FVIIa)	RRVA QVI230I PSTYV	15.38 %	38.46 %
Human factor IXa (FIXa)	RNVI RII254P HHNYN	15.38 %	38.46 %
Human factor Xa (FXa)	EAVH EVE266VVIKHNRFT	25.00 %	37.50 %
Human protein S (PS)	---------------------	----------	-----------

[a] Identical amino acid residues are highlighted in green, similar amino acid residues are displayed in yellow. The original sequence of HBM **4** in human PZ is shown in purple. [b] Central amino acid residues are numbered according to the mature proteins.

Table S4. Sequence alignments of the region around HBM **4** in human PZ with other vitamin K-dependent proteins of the human blood coagulation system.

Proteins	HBM 1 in APC[a],[b]	HBM 2 in APC[a],[b]
Human activated protein C (APC)	WIHG**H**^{391}IRDK	TGWG**Y**^{289}HSSR
Human protein Z (PZ)	-----------	SGWAR^{265}NGTD

[a] Identical amino acid residues are highlighted in green, similar amino acid residues are displayed in yellow. The original sequences of HBM **1** and HBM **2** in human APC are shown in blue. [b] Central amino acid residues are numbered according to the mature proteins.

WILEY-VCH
